# COVID-19-Associated Pneumatocele and Subsequent Pneumothorax

**DOI:** 10.7759/cureus.36692

**Published:** 2023-03-26

**Authors:** Phyoe Kyaw Pyae, Muhammad Arif

**Affiliations:** 1 Acute Medicine, Watford General Hospital, Watford, GBR

**Keywords:** covid-19 mortality, covid-19 complications, covid-19 retro, pneumothorax, pneumatocele

## Abstract

We report a case of pneumatocele and subsequent pneumothorax, 20 days after being treated for coronavirus disease 2019 (COVID-19) and discharged. This 64-year-old patient was initially treated for COVID-19 pneumonia and pulmonary embolism (PE) over a two-week-long admission. He was discharged and then re-presented two days post-discharge with sudden exacerbation of breathlessness. Blood tests showed worsening inflammatory markers likely associated with bacterial infection, and imaging revealed multiple pneumatoceles and subsequent pneumothorax. Unfortunately, he rapidly deteriorated and passed away. This case report adds to the growing concern in the literature about the serious and life-threatening complications of COVID-19 infection and raises awareness of this rare complication.

## Introduction

The overwhelming burden of the coronavirus disease 2019 (COVID-19) pandemic on healthcare systems around the world and the associated morbidity and mortality is well known and widely reported. In addition to managing the treatment of COVID-19 infection, its associated disease course, and host inflammatory response, it is important to monitor and carefully manage the complications associated with the disease and the treatment such as ventilation-associated lung injury, steroid-induced complications and so on. Pneumomediastinum and pneumothorax are rare complications of COVID-19 infection, with an overall incidence of 0.97% [1], especially in the absence of risk factors, ventilation-associated lung injury, or underlying respiratory conditions [2]. In immunocompetent patients with no previous respiratory conditions, only Staphylococcus and fungal pneumonia are recognised infective causes of free air leak [3]. It is becoming more recognisable that spontaneous air leaks in the form of pneumothorax, pneumomediastinum and pneumatocele are associated with COVID-19 infection and the risk increases with male sex, smoking history, underlying lung disease and invasive ventilation [1]. We report a case of a 64-year-old gentleman who developed pneumatocele and subsequent pneumothorax after completing treatment for COVID-19 pneumonia.

## Case presentation

A 64-year-old male patient presented with sudden worsening of shortness of breath and hypoxia, two days after discharge from the hospital after being treated for COVID-19 pneumonia and pulmonary embolism (PE). He was an ex-smoker with a 30-pack-year smoking history, fit and independent, with no past medical history or regular medication.

The previous admission was for COVID-19 pneumonia and it was two-week long, during which his oxygen requirement increased from a 35% venturi mask (VM) to a 15-litre non-breather mask (NRM). Then, one day after being on high-flow nasal oxygen (HFNO), he required escalation to ventilation with continuous positive airway pressure (CPAP). He was given tocilizumab treatment the day before starting HFNO to reduce the risk of requiring intubation. He was also managed with proning and 10 days of dexamethasone therapy, which was then switched to a prednisolone weaning regimen over the next six weeks due to evidence of significant pulmonary infiltrates. Repeat procalcitonin was found to be high, and he was started on antibiotics to complete a two-week course. He improved eventually and was discharged with home oxygen (2-3 litres/hour) and rivaroxaban for PE, to be followed up in a virtual COVID hospital.

**Figure 1:**
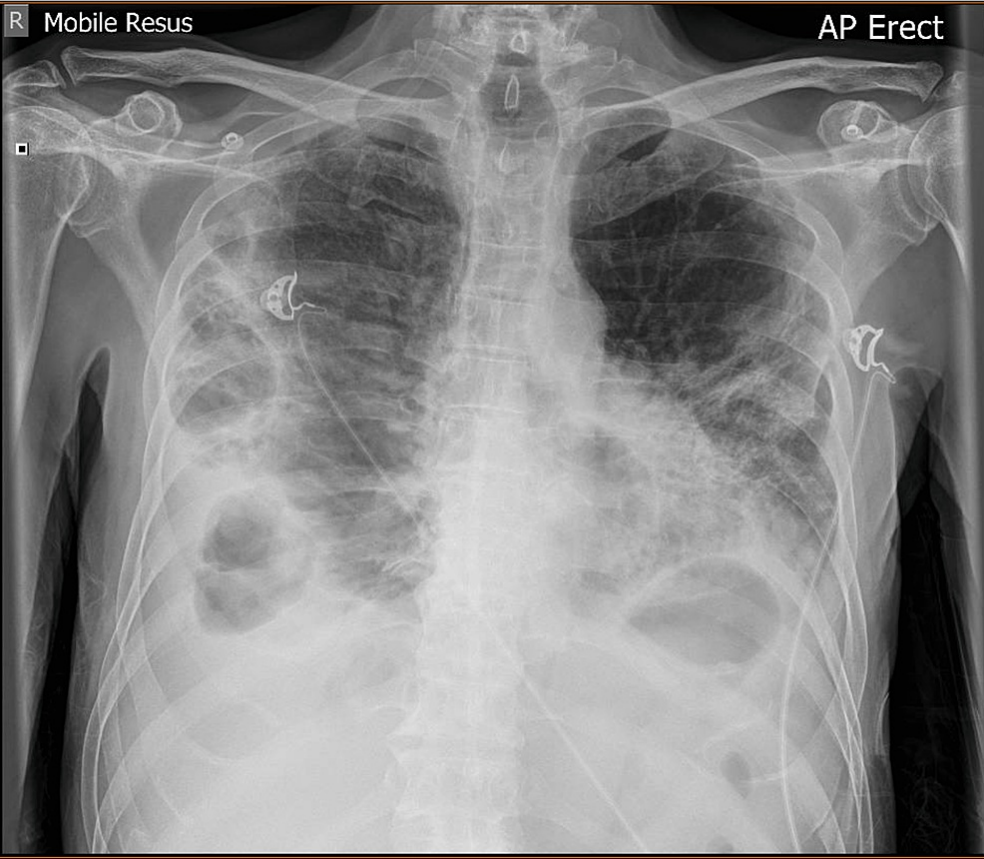
Chest X-ray on day 0 of current admission

**Figure 2:**
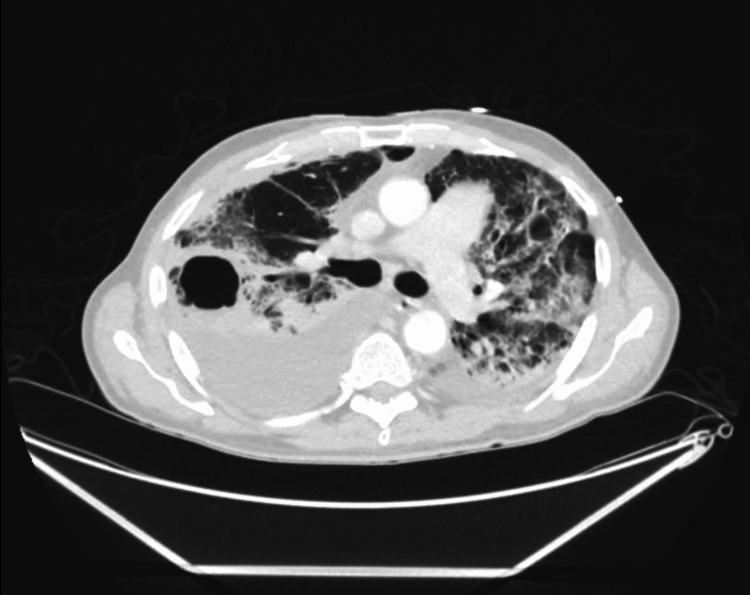
CT thorax on day 0 of current admission

**Figure 3:**
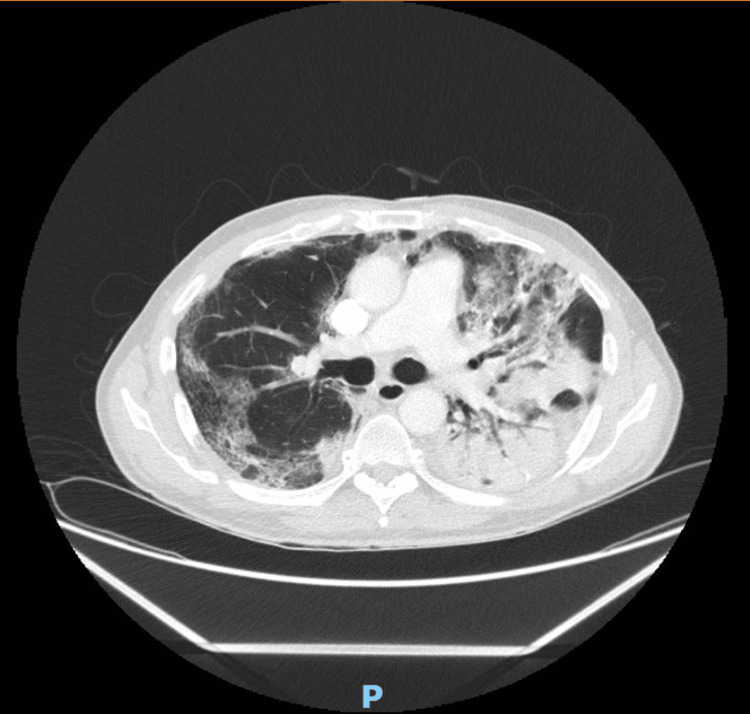
CT thorax previous admission four days before discharge

**Figure 4:**
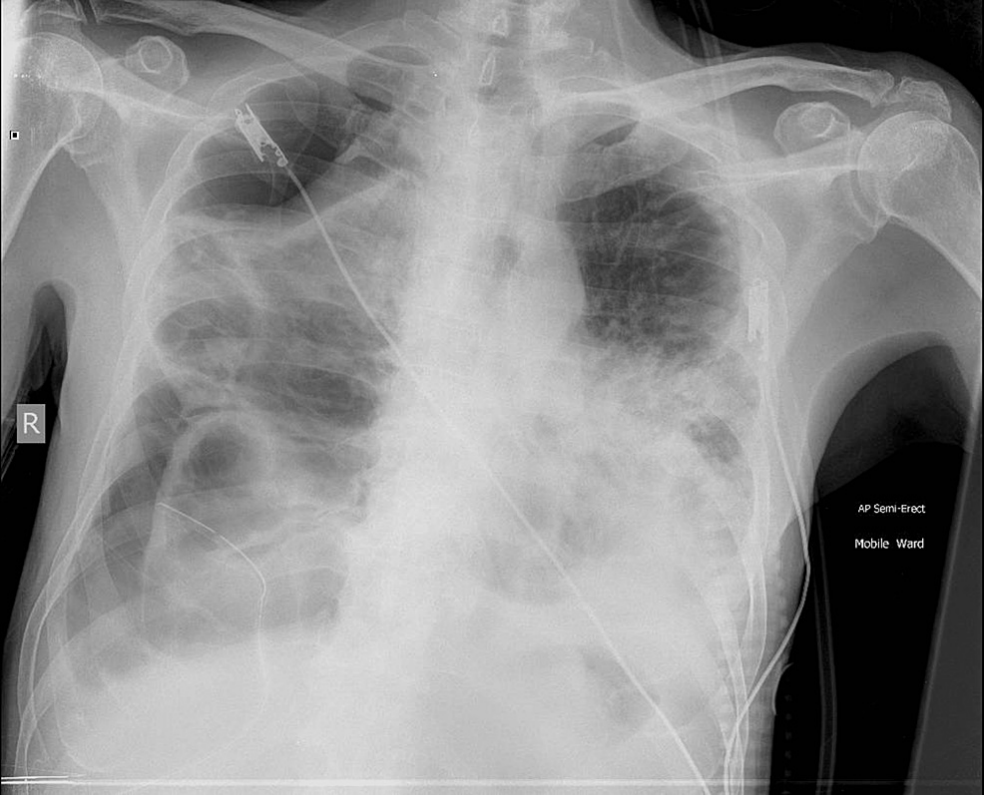
One hour post chest drain insertion chest X-ray on day 1 of current admission

Two days after discharge, he became acutely short of breath again and presented in the accident and emergency department. Blood test results are illustrated in Table 1. A chest X-ray showed extensive bilateral shadowing with multiple cavitations, consistent with COVID-pneumonia, with an increase in right basal effusion (Figure 1). A computerized tomogram thorax scan (CT thorax) reported a large right pleural effusion with a collapse of the right lower lobe and a large pneumatocele in the anterolateral aspect of the collapsed right lower lobe measuring 5.4 cm x 4.3 cm x 5.6 cm, with another septated pneumatocele in the lateral aspect of the posterior segment of the right upper lobe measuring 4.3 cm x 3.9 cm x 6.8 cm (Figure 2). These were not present in the previous CT performed on previous admission (Figure 3), consistent with newly developed pulmonary parenchymal destruction. There were extensive alveolar and ground glass opacities and honeycombing and crazy paving in the rest of both lungs. He was admitted to the COVID-respiratory ward under close monitoring. However, within 24 hours into admission, the patient became increasingly breathless and desaturated, an emergency chest drain was inserted, and he was started on bi-level positive pressure ventilation (BiPAP). Repeat chest X-ray showed right-sided pneumothorax (Figure 4). Unfortunately, the patient deteriorated and passed away the following day. Pleural aspirate results showed lactate dehydrogenase (LDH) 1066 U/L, total protein 38 g/L, cultures negative, and acid-fast bacilli were not seen. Cytology showed aggregates of neutrophils and macrophages, supporting this being a reactive effusion. Total serum immunoglobulin E (IgE), aspergillus IgE and IgG were normal.

**Table 1:** Biochemistry results in cumulative chronological comparison order

	Previous admission			Current admission
	2 weeks before discharge	1 week before discharge	1 day before discharge	
White cell count	4.20	6.30	28.90	27.90
Neutrophils	2.78	5.51	27.17	26.11
Lymphocytes	0.99	0.58	0.72	0.70
C-reactive protein	43	<5.0	11.5	229
Procalcitonin	0.07	0.06	0.34	0.52

## Discussion

The most common radiological presentation of COVID-19 is the development of ground-glass opacities with or without patchy consolidations. There have also been a few reports of pneumomediastinum, pneumothorax and pneumatoceles [1-4]. It could be due to the observation that COVID-19 tends to affect peripheral and sub-pleural distribution. On the other hand, it could also be secondary to the effect of airway pressure from ventilation such as CPAP.

Spontaneous pneumothorax is a rare but life-threatening complication as seen in the case presented and reported by other studies as a 1-2% cause of COVID-19-related death [5-6]. Although in some cases, resolution of pneumothorax was achieved with either conservative management or chest drain insertion [7-8], it could be challenging with the presence of acute respiratory distress syndrome (ARDS). Management would then depend on the location and size of the lesions, as well as the clinical stability of the patient. In this case, the patient deteriorated rapidly within 24 hours of admission. It led to an emergency chest drain being inserted, without which the deterioration could continue. Although it did not change the course of the deterioration nor prevent mortality, it was the most suitable intervention at that time based on risk and benefit analysis.

From the histologic perspective, it has been reported from the post-mortem examination of severe acute respiratory syndrome patients that diffuse alveolar damage leads to dilated cystic airspaces with a honeycomb appearance, which predisposes to air leaks secondary to rupture of the lesions [7]. This histopathological finding could account for the development of pneumatocele and subsequent pneumothorax in our patient as a complication of COVID-19 pneumonia.

In terms of pneumatocele formation, various theories have been described regarding the pathophysiology: pulmonary overinflation, drainage of necrotic lung parenchyma with subsequent enlargement, focal air collection due to inflammation and necrosis of the airway wall in the interstitial tissue [9]. A pneumatocele often resolves with conservative management due to its transient nature and persists at most for a few months [9]. Complications include local compression, infection, rupture and pneumothorax [9]. In this case, the pneumatocele found on admission did not appear to include the air-fluid level although consolidation was seen in the lung parenchyma outside the pneumatocele, suggestive of persistent pneumonia from COVID-19 infection or superimposed bacterial infection. Unfortunately, it subsequently ruptured and formed a pneumothorax with rapid clinical deterioration and cardiorespiratory compromise.

In addition, during the course of treatment of COVID-19 pneumonia in the previous recent hospital admission, this patient received HFNO and then CPAP to achieve adequate ventilation. Given that pneumothorax is known to be a complication of ventilator-induced lung injury and pulmonary overinflation as a possible cause of pneumatocele, it is important to choose the treatment modality carefully when escalating ventilation to either HFNO or CPAP when NIV (non-invasive ventilation) is required as well as adjusting and controlling the pressure setting on CPAP.

## Conclusions

Pneumomediastinum, pneumothorax and pneumatoceles are some of the rare but serious complications of COVID-19 disease associated with high mortality and concomitant use of high-flow nasal oxygen (HFNO) and non-invasive ventilation (NIV). Early recognition of this complication is important, as it is associated with very high mortality. While in some cases of COVID pneumonitis, the use of HFNO and high-pressure settings in CPAP may be unavoidable, the risk of ventilation-associated lung injury, including pneumothorax and pneumatocele, must be kept in mind.
